# CULTURAL COMPETENCE TRAINING FOR HOSPICE STAFF: FINDINGS FROM A NATIONAL HOSPICE SURVEY

**DOI:** 10.1093/geroni/igz038.019

**Published:** 2019-11-08

**Authors:** Nathan Boucher, Kimberly S Johnson

**Affiliations:** 1 Durham VA Health System, Durham, North Carolina, United States; 2 Duke University, Durham, North Carolina, United States

## Abstract

Compared to whites, racial/ethnic minorities are less likely to enroll in hospice and if they enroll, more likely to experience poor quality care. Building cultural competence (CC) among hospice staff is a strategy that may reduce these disparities. We conducted a national survey of hospices’ practices to promote CC. A total of 197 hospices participated; most were not-for-profit (80%) with an average daily census over 100 (53%); 73% offered staff cultural competence training (CCT). There were no differences in characteristics of hospices who offered CCT and those that did not. Of hospices offering CCT, 54% held it annually. Most trainings were one hour (60%); content was delivered via web (58%) and/or lecture (57%). While over 90% of staff (i.e., nurses, social workers, and chaplains) completed CCT, a smaller proportion of medical directors (63%), senior leaders (70%) and board members (23%) did so. Most common (>70%) topics were: cross-cultural communication, death and illness beliefs, and spirituality’s role, and healthcare disparities. The majority focused on African-Americans (83%), Hispanics (76%), and Asians (61%)—the most common U.S. minority groups. Almost 30% reported no assessment of effectiveness of CCT while 45% reported a quiz at the end. In this study, most hospices offered some CCT. CCT has been shown to improve healthcare providers’ knowledge and skills in caring for diverse patients and is associated with increased patient satisfaction. Future research should evaluate effectiveness of CCT in improving the ability of hospices to deliver high quality end-of-life care to diverse groups of older adults.

